# Chemokines CCL3/MIP1α, CCL5/RANTES and CCL18/PARC are Independent Risk Predictors of Short-Term Mortality in Patients with Acute Coronary Syndromes

**DOI:** 10.1371/journal.pone.0045804

**Published:** 2012-09-21

**Authors:** Saskia C. A. de Jager, Brenda W. C. Bongaerts, Michael Weber, Adriaan O. Kraaijeveld, Mat Rousch, Stefanie Dimmeler, Marja P. van Dieijen-Visser, Kitty B. J. M. Cleutjens, Patty J. Nelemans, Theo J. C. van Berkel, Erik A. L. Biessen

**Affiliations:** 1 Division of Biopharmaceutics, Leiden Amsterdam Centre for Drug Research, Leiden University, Leiden, The Netherlands; 2 Department of Pathology, Cardiovascular Research Institute Maastricht, Maastricht University Medical Centre, Maastricht, Maastricht, The Netherlands; 3 Department of Cardiology, Kerckhoff Heart Centre, Bad Nauheim, Germany; 4 Department of Cardiology, Leiden University Medical Centre, Leiden, The Netherlands; 5 Institute for Cardiovascular Regeneration, Centre of Molecular Medicine, Goethe-University Frankfurt, Frankfurt am Main, Germany; 6 Department of Clinical Chemistry, Maastricht University Medical Centre, Maastricht, The Netherlands; 7 Department of Epidemiology, Maastricht University, Maastricht, The Netherlands; South Texas Veterans Health Care System and University Health Science Center San Antonio, United States of America

## Abstract

Cytokines play an important role in ischemic injury and repair. However, little is known about their prognostic value in cardiovascular disease. The aim of this study was to investigate the prognostic importance of chemokines CCL3/MIP-1α, CCL5/RANTES and CCL18/PARC for the risk of future cardiovascular events in patients with acute coronary syndromes (ACS). Baseline levels of CCL3/MIP-1α, CCL5/RANTES and CCL18/PARC were determined in ACS patients from the Bad Nauheim ACS II registry (n = 609). During the following 200 days, patients were monitored for the occurrence of fatal and non-fatal cardiovascular events. Patients with CCL3/MIP1α, CCL5/RANTES and CCL18/PARC concentrations in the highest tertile were associated with an increased risk of a fatal event during follow-up (HR: 2.19, 95%CI: 1.04–4.61 for CCL3/MIP1α, HR: 3.45, 95%CI: 1.54–7.72 for CCL5/RANTES and HR: 3.14, 95%CI: 1.33–7.46 for CCL18/PARC). This risk was highest for patients with all three biomarkers concentrations in the upper tertile (HR: 2.52, 95%CI: 1.11–5.65). Together with known risk predictors of cardiovascular events, CCL3/MIP-1α, CCL5/RANTES and CCL18/PARC combined improved the c-statistics from 0.74 to 0.81 (p = 0.007). In conclusion, CCL3/MIP-1α, CCL5/RANTES and CCL18/PARC are independently associated with the risk of short-term mortality in ACS patients. Combining all three biomarkers further increased their prognostic value.

## Introduction

Cardiovascular diseases continue to be a major cause of morbidity and mortality in Western societies [1]. Clinically evident cardiovascular disease is generally attributable to atherothrombosis and often manifests itself by acute coronary syndromes, such as unstable angina pectoris (UAP) and acute myocardial infarction (AMI). ACS patients have a considerably increased risk of secondary cardiovascular events during follow-up, including recurrent ischemia, myocardial (re)infarction, stroke, embolization and (re)stenosis-related stable angina pectoris. For adequate treatment and patient monitoring it is pivotal to distinguish between individuals with a low and a high risk of secondary events. This risk stratification has long relied on demographic and other classic patient factors [2], [3], but is now increasingly supported by several biomarkers, such as troponin T (TnT) and C-reactive protein (CRP) [4], [5]. Still, the markerś performance of predicting future risk at the level of the patient is only moderate and warrants further investigation into new prognostic biomarkers.

Chemokines, a class of chemotactic cytokines, come into play in response to acute cardiovascular events, coordinating inflammation, necrosis, neovascularisation and leukocyte recruitment [6], [7], [8]. Amongst other processes, leukocyte recruitment and infiltration are vital at all stages of atherosclerosis [9]. It has been shown that several chemokines, including CCL3/MIP1a, CCL5/RANTES, and CCL18/PARC, are expressed in atherosclerotic lesions [10], [11], [12], [13]. CCL5/RANTES is produced by various leukocyte subsets in response to inflammatory stimuli, such as monocytes, macrophages and T cells, but also smooth muscle cells [9], [14]. CCL3/MIP1α and CCL5/RANTES are released by activated platelets and are implied to contribute to attraction of leukocytes in atherothrombosis [7], [15]. Indeed genetic deletion of CCR5, an important receptor for CCL3/MIP1α and CCL5/RANTES, protected atherosclerotic prone mice from atherosclerosis and appeared to be associated with impaired Th1 immunity [16], [17]. Inflammatory stimuli were recently shown to induce CCL3/MIP1α-mediated neutrophil migration towards sites of inflammation. In turn, circulating neutrophil numbers have been reported to be a prognostic factor for future cardiovascular events [18], [19]. CCL18/PARC has specific chemotactic activity on T cells and this chemokine can activate fibroblasts, thereby directly contributing to lung fibrosis and possibly myocardial fibrosis upon ischemia [20]. Furthermore, various chemokines have been suggested to play a direct role in post-ischemic injury repair after AMI, not only directly - by mediating the recruitment of neutrophils, mast cells and stem cells to the lesion - but also indirectly - by modulating necrosis and angiogenesis [21], [22], [23], [24], [25], [26], [27].

In only few (genetic) epidemiological studies chemokine concentrations have been studied in association with ACS [28], [29], [30], [31], [32]. The most elaborately studied chemokines in atherosclerosis, CCL2/MCP-1 and CCL5/RANTES, have been implied to reflect the burden of atherosclerotic lesions in cardiovascular disease patients [30], [32]. Different genetic polymorphisms for CCL5/RANTES were identified as either harmful or protective for the development of cardiovascular disease [31], [33], [34], [35]. Recently, we reported elevated serum levels of CCL5/RANTES and CCL18/PARC in a small patient cohort consisting of subjects with unstable angina pectoris and both chemokines were identified as markers of refractory UAP [32]. In a similar patient population we showed that CCL3/MIP-1α levels were highly elevated in patients with AMI and with UAP. In addition, we observed that CCL3/MIP-1α was a prognostic factor of future cardiovascular events [36].

In an effort to study the potential of chemokine markers for risk stratification in ACS, we examined the prognostic value of baseline levels of CCL3/MIP-1α, CCL5/RANTES and CCL18/PARC in a prospective cohort of 762 ACS patients. The occurrence of both fatal and non-fatal cardiovascular events was assessed after a follow-up period of 200 days. We hypothesized that high blood concentrations of these biomarkers are associated with an increased risk of adverse cardiovascular outcomes.

## Methods

### Ethics Statement

This study complies with the guidelines in the Declaration of Helsinki. Ethics approval was granted by the regional ethics committee. All participants gave full informed written consent, which included consent for biomarker analysis prior to inclusion into the study.

### Study Population

Between April 2005 and November 2006, all consecutive patients admitted to the Kerckhoff Heart Centre in Bad Nauheim (Germany) were recruited for the Bad Nauheim ACS II registry. Patients, on suspicion of acute coronary syndromes (ACS) with chest pain complaints within the last 48 hours and referred for early coronary angiography or primary percutaneous intervention (PCI), were eligible for inclusion (n = 762). Admission took place either directly through the emergency medical system or through transferral from community hospitals. Patients with multi-vessel disease and cardiogenic shock were excluded from inclusion. A diagnosis of UAP, non-ST elevation myocardial infarction (NSTEMI) or STEMI was made by the treating physician based on the electrocardiogram (ECG) in combination with serial TnT measurement as defined by the European Society of Cardiology/American College of Cardiology guidelines from the year 2000 [37]. Blood was drawn upon admission, prior to angiography and revascularization procedures. Pre-treatment with clopidogrel or a GP IIb/IIIa inhibitor was left to the discretion of the treating physician. Each patient provided information concerning medical history and medication regime. Baseline ECG characteristics were recorded, the presence or absence of several cardiovascular risk factors was assessed and all patients were subjected to a physical examination. In addition, time from the onset of symptoms until the time of first blood withdrawal was ascertained.

All patients were monitored for 200 days. Follow-up end points were a new ACS (e.g. cardiac ischemia and AMI) and coronary revascularisation (PCI and CABG), which were combined as non-fatal events. The fatal events comprised all cases of all-cause mortality. Patients were considered to be at risk for a fatal or non-fatal event from the date of entry into the study until the date at which the event occurred. Otherwise, follow-up ended at the date of withdrawal from the study or at 200 days after entry.

**Figure 1:**
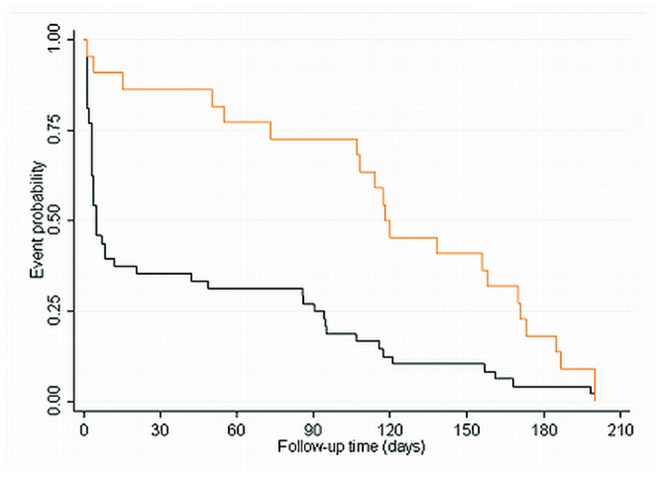
Kaplan-Meier curves for the occurrence of fatal and non-fatal events in patients with acute coronary syndromes of the Bad-Nauheim ACS II registry. P<0.001 for the comparison of both event functions by the log-rank test.

### Biochemical Analyses

Baseline blood specimens were centrifuged within one hour after collection and the serum was snap frozen and stored at −70°C until further analyses. Samples were available for 706 (93%) patients.

Baseline serum levels of CCL3/MIP-1α, CCL5/RANTES and CCL18/PARC were determined by commercially available ELISA kits according to the manufacturers’ protocol. ELISA kits were obtained from Invitrogen (Breda, the Netherlands; human RANTES/CCL5 ELISA kit, ≤4.7% intra-assay variation) and R&D systems (Abingdon, United Kingdom; Human CCL3/MIP-1 alpha Quantikine ELISA Kit, ≤8.9% intra-assay variation and Human CCL18/PARC DuoSet). Cardiac TnT was measured by electrochemiluminescence immunoassay (third generation for TnT, <10%CV, Elecsys, Roche Diagnostics, Mannheim, Germany). NT-proBNP was assessed by electrochemiluminescence immunoassay (Elecsys proBNP (≤2.7% intra-assay variation) Roche Diagnostics). hsCRP was measured using a near-infrared particle immunoassay rate method (<5% intra-assay variation) with the use of the Beckman LX-20pro (Beckman Coulter, Inc). Creatine Kinase isoenzyme MB was determined by an *in vitro* immuno-inhibition assay (<10% intra-assay variation) on a Roche/Hitachi analyzer (Creatine Kinase MB liquid, Roche Diagnostics).

**Table 1:** Baseline characteristics and biomarker levels of the study population according to the occurrence of a cardiovascular event within six months of follow-up, Bad Nauheim ACS II registry.

	Event during follow-up		
	No (n = 539)	Yes (n = 70)	*P*-value
**Baseline characteristics**			
Age^1^– years	64±13	66±12	0.29
Gender (% male)	70	73	0.62
Body mass index^1^– kg/m^2^	27.9±4.4	27.8±6.3	0.83
Hypertension (% yes)	66	60	0.29
Hyperlipidemia (% yes)	41	33	0.21
Diabetes (% yes)	18	34	<0.01
Smoking (% yes)	32	26	0.28
Family history of CVD (% yes)	21	9	0.02
History of AMI (%yes)	10	16	0.15
Prior revascularization procedure (% yes)	15	19	0.83
Blood drawing^3,5^– hrs	5.9 (2.5–13.9)	5.0 (2.3–10.3)	0.93
**Baseline biomarkers levels** 2			
CCL3/MIP-1α – pg/ml	25.8 (14.9–36.5)	31.4 (17.2–42.2)	0.09
CCL5/RANTES – ng/ml	25.4 (12.3–39.7)	30.7 (14.2–48.2)	0.13
CCL18/PARC – ng/ml	54.0 (36.3–80.5)	66.9 (43.3–104.8)	<0.01
TnT – ng/ml	0.21 (0.04–0.78)	0.29 (0.06–0.90)	0.12
hsCRP – mg/l	3.07 (1.37–7.70)	7.40 (1.89–19.8)	<0.01
NT-proBNP – pg/ml	523 (156–1805)	1326 (305–5549)	<0.01
CK-MB – ng/ml	12.8 (4.5–40.9)	11.3 (4.5–48.1)	0.61
CK-MB max^4^– ng/ml	174 (0.00–834)	269 (0.00–1307)	0.36
Creatinine – mg/dl	0.89 (0.77–1.08)	1.15 (0.84–1.42)	<0.01
**Patient status upon admission**			
UAP (%)	10.6	13.9	0.32
STEMI (%)	55.4	58.3	0.71
NSTEMI (%)	33.5	29.2	0.51
Killip Class ≥2 (% yes)	7	7	0.93
Right coronary artery stenosis (% yes)	6	17	<0.01
Left coronary artery stenosis (% yes)	45	41	0.58
ST-segment elevation (% yes)	9	4	0.16
T-segment inversion (% yes)	5	9	0.22
Left ventricular ejection fraction^1,6^ (%)	48±11	44±13	0.04

CVD: cardiovascular disease; AMI: acute myocardial infarction; TnT: Troponin T; CRP: C-Reactive Protein; NT-proBNP: N-terminal pro-Brain Natriuretic Peptide; CK-MB: Creatinine Kinase-MB; UAP: unstable angina pectoris; STEMI: ST-segment elevated myocardial infarction; NSTEMI: Non-ST-segment elevated myocardial infarction.

1 Presented as mean ± sd,

2 Presented as median (interquartile range),

3 Time of blood drawing since onset of symptoms,

4 based on values of 513 event free patients and 68 event patients,

5 based on values of 516 event free patients and 63 event patients,

6 based on values of 480 event free patients and 53 event patients.

### Statistical Analysis

Data analysis was based on 609 subjects with complete information available on biomarker concentrations and other baseline patient characteristics. Patients with and without a cardiovascular event during follow-up were compared for differences in baseline characteristics. Regarding nominal variables, we used the χ^2^ test for comparisons of proportions. For continuous variables with a normal distribution we applied the Student’s t-test for independent samples or analysis of variance (ANOVA). For continuous variables that were not normally distributed, we used nonparametric Kruskal-Wallis test.

Associations between chemokine levels and the risk of future cardiovascular events, hazard ratios (HR) and 95% confidence intervals (CI) were estimated using Cox proportional hazards models. The proportional hazards assumption was tested using the scaled Schoenfeld residuals [38] Chemokine concentrations were categorized into tertiles of which the lowest tertile was considered as the reference category. Confounding effects of age at baseline (years), sex, body mass index (bmi) (kg/m^2^), hypertension (yes/no), hyperlipidemia (yes/no), diabetes (yes/no), smoking (yes/no), a positive family history of cardiovascular disease (yes/no), ST-segment depression present on baseline ECG (yes/no), previous AMI (yes/no), previous revascularization (yes/no), and baseline levels NT-proBNP (pg/ml), CKMB (ng/ml), hsCRP (mg/l) and TnT (ng/ml) were evaluated for their effect on the relationship between chemokine concentrations and risk of future events. Those variables that altered the estimate for the exposure coefficient between statistical models with and without the potential confounder by more than 10% were included in the analyses. Both univariate and multivariable Cox models were performed for fatal and non-fatal events combined, as well as for both endpoints separately.

**Table 2:** Hazard Ratios (95% confidence intervals) for a future cardiovascular event1 during 200 day follow-up in patients with the acute coronary syndrome, according to baseline levels of CCL3/MIP-1α, CCL5/RANTES and CCL18/PARC, Bad Nauheim ACS II registry.

Tertiles of biomakers^2^	Event during follow-up			
	No (n = 539)	Yes (n = 70)	Univariate HR(95% CI)	Multivariate HR^3^ (95% CI)
**CCL3/MIP-1α**				
Tertile 1 (low)	187	21	1.00 (Reference)	1.00 (Reference)
Tertile 2	189	18	0.83 (0.44–1.55)	0.92 (0.49–1.75)
Tertile 3 (high)	163	31	1.67 (0.97–2.94)	1.60 (0.90–2.83)
p-value for trend			0.267	0.279
**CCL5/RANTES**				
Tertile 1 (low)	177	20	1.00 (Reference)	1.00 (Reference)
Tertile 2	182	17	0.86 (0.45–1.64)	1.10 (0.56–2.18)
Tertile 3 (high)	180	33	1.63 (0.93–2.84)	1.99 (1.10–3.61)
p-value for trend			0.009	0.001
**CCL18/PARC**				
Tertile 1 (low)	185	17	1.00 (Reference)	1.00 (Reference)
Tertile 2	179	21	1.29 (0.68–2.44)	1.49 (0.78–2.85)
Tertile 3 (high)	175	32	1.88 (1.05–3.39)	1.81 (0.98–3.32)
p-value for trend			<0.001	0.001

HR: hazard ratio; CI: confidence interval.

1 A cardiovascular event is defined as the occurrence of death, an acute myocardial infarction or an urgent revascularization procedure.

2 Tertile boundaries for CCL3/MIP-1α: 18.7–33.3 pg/ml, CCL5/RANTES: 16.2–134.15 ng/ml, CCL18/PARC: 43.0–70.9 ng/ml.

3 Adjusted for age, sex, diabetes, smoking, family history of cardiovascular disease and baseline levels of NT-proBNP, CK-MB and TnT.

In order to evaluate the added prognostic value of the chemokines on top of that provided by conventional predictors of future cardiovascular events, receiver-operating characteristic (ROC) curves were constructed for each endpoint, using the predicted values from multivariable regression models with and without the studied chemokines. Since standard models to derive ROC curves do not exist for survival data, we used logistic regression models with a dichotomous clinical endpoint as dependent variable (yes/no event during follow-up). The area under the ROC curve (AUC), or c-statistic, were compared according to the method described by Hanley et al. [39]. This method accounts for the fact that the c-statistic from the predicted values from the models with and without the chemokines were derived from the same sample of patients.

Two-sided *P*-values <0.05 were considered to indicate statistical significance. All analyses were performed with the STATA statistical software package (version 10.0).

**Table 3:** Hazard Ratios (95% confidence intervals) for a fatal future cardiovascular event during follow-up in patients with the acute coronary syndrome, according to baseline levels of CCL3/MIP-1α, CCL5/RANTES and CCL18/PARC, Bad Nauheim ACS II registry.

	Fatal event during follow-up				Non-fatal event during follow-up			
Tertiles of biomakers^1^	No (n = 539)	Yes (n = 48)	Univariate HR (95% CI)	Multivariate HR^2^ (95% CI)	No (n = 539)	Yes (n = 22)	Univariate HR (95% CI)	Multivariate HR^2^ (95% CI)
**CCL3/MIP-1α**								
Tertile 1 (low)	187	11	1.00 (Reference)	1.00 (Reference)	187	10	1.00 (Reference)	1.00 (Reference)
Tertile 2	189	13	1.14 (0.51–2.54)	1.30 (0.57–2.98)	189	5	0.47 (0.16–1.37)	0.48 (0.16–1.41)
Tertile 3 (high)	163	24	2.44 (1.20–4.99)	2.19 (1.04–4.61)	163	7	0.83 (0.31–2.17)	0.88 (0.33–2.34)
p-value for trend			0.103	0.123			0.605	0.657
**CCL5/RANTES**								
Tertile 1 (low)	177	11	1.00 (Reference)	1.00 (Reference)	177	9	1.00 (Reference)	1.00 (Reference)
Tertile 2	182	11	0.98 (0.43–2.26)	1.58 (0.63–3.98)	182	6	0.68 (0.24–1.91)	0.66 (0.23–1.89)
Tertile 3 (high)	180	26	2.25 (1.11–4.56)	3.45 (1.54–7.72)	180	7	0.81 (0.30–2.17)	0.78 (0.29–2.13)
p-value for trend			<0.001	<0.001			0.258	0.249
**CCL18/PARC**								
Tertile 1 (low)	185	7	1.00 (Reference)	1.00 (Reference)	185	10	1.00 (Reference)	1.00 (Reference)
Tertile 2	179	17	2.46 (1.02–5.94)	3.07 (1.25–7.53)	179	4	0.42 (0.13–1.35)	0.45 (0.14–1.45)
Tertile 3 (high)	175	24	3.42 (1.47–7.94)	3.14 (1.33–7.46)	175	8	0.80 (0.32–2.04)	0.84 (0.31–2.23)
p-value for trend			<0.001	<0.001			0.860	0.963

HR: hazard ratio; CI: confidence interval.

1 Tertile boundaries for CCL3/MIP-1α: 18.7–33.3 pg/ml, CCL5/RANTES: 16.2–134.15 ng/ml, CCL18/PARC: 43.0–70.9 ng/ml.

2 Adjusted for age, sex, diabetes, smoking, family history of cardiovascular disease and baseline levels of NT-proBNP, CK-MB and TnT.

## Results

Of the total study population, 609 patients (80%) had complete information on baseline biomarker concentrations, traditional risk predictors for cardiovascular disease and other covariates. Average follow-up time was 189 days (189±14.1 days) during which a total of 70 patients suffered an event. Figure 1 shows Kaplan Meier curves for the fatal (n = 48) and the non-fatal events (n = 22) during follow-up. The majority of the fatal events had occurred in patients shortly after admission. Comparison of both event functions with the log-rank test showed that fatal events occurred significantly earlier during follow-up than non-fatal events (p<0.05).

Baseline characteristics of the study population are presented in table 1 according to the occurrence of an adverse event during follow-up. Compared with the 539 patients who remained event-free during follow-up, the event population was enriched in patients with diabetes and left coronary artery stenosis, while a family history of cardiovascular disease was seen less frequently. Median serum concentrations of CCL18/PARC, hsCRP, NT-proBNP and creatinine were significantly higher in patients with an event than in patients without an event (p<0.01).

Being important measures of the degree of myocardial damage, left ventricular ejection fraction and end diastolic diameter and -mass were comparable between UAP, NSTEMI and STEMI patients (data not shown).

**Table 4:** Hazard Ratios (95% confidence intervals) for a future cardiovascular event^1^ during follow-up in patients with the acute coronary syndrome, according to the number of chemokines (CCL3/MIP-1α, CCL5/RANTES and CCL18/PARC) in the highest tertile, Bad Nauheim ACS II registry.

Nr. of chemokines in thehighest tertile	Fatal and non-fatal events during follow-up combined				Fatal events during follow-up			
	No (n = 539)	Yes (n = 70)	Univariate HR (95% CI)	Multivariate HR^2^ (95% CI)	No (n = 562)	Yes (n = 47)	Univariate HR (95% CI)	Multivariate HR^2^ (95% CI)
None^3^	180	12	0.49 (0.25–0.96)	0.45 (0.23–1.11)	187	5	0.27 (0.12–0.83)	0.29 (0.10–0.85)
At least one	223	31	1.00 (Reference)	1.00 (Reference)	233	21	1.00 (Reference)	1.00 (Reference)
Two out of three	113	16	1.03 (0.57–1.89)	1.16 (0.46–2.17)	117	12	1.14 (0.56–1.76)	1.07 (0.51–2.22)
All three	23	11	3.11 (1.56–6.18)	2.71 (1.14–4.91)	25	9	3.12 (1.31–7.40)	2.52 (1.11–5.65)
p-value for trend			0.038	0.018			0.020	0.011

HR: hazard ratio; CI: confidence interval.

1 A cardiovascular event is defined as the occurrence of death, an acute myocardial infarction or an urgent revascularization procedure.

2 Adjusted for age, sex, diabetes, smoking, family history of cardiovascular disease and baseline levels of NT-proBNP, CK-MB and TnT.

3 All three chemokines concentrations in the lowest tertile.

In Table 2, results are presented for the relationship between the chemokines under study and the risk of adverse events during follow-up (fatal and non-fatal events combined). We divided biomarker concentrations into tertiles to avoid creating too small subgroups given the relatively low number of follow-up events. Univariate and multivariate HRs were highly similar. Compared to patients in the lowest tertile of CCL5/RANTES and CCL18/PARC, patients in the highest tertile were associated with an increased risk of adverse events. A similar observation was seen for patients in the highest tertile of CCL3/MIP-1α, albeit statistical significance was not reached.

When performing analyses for fatal and non-fatal end points separately (table 3), we observed positive associations between the chemokine levels and risk of future fatal events. Patients in the highest tertile of CCL3/MIP-1α, CCL5/RANTES and CCL18/PARC had a 2 to 3.4-fold higher risk of mortality during follow-up, compared to patients in the lowest tertiles. A statistically significantly linear trend was observed over the tertiles of CCL5/RANTES and CCL18/PARC (p<0.001). No relationship was apparent for the three chemokines and the risk of non-fatal events. The number of cases in these subgroups was very small and warrants caution when interpreting the risk estimates.

**Figure 2:**
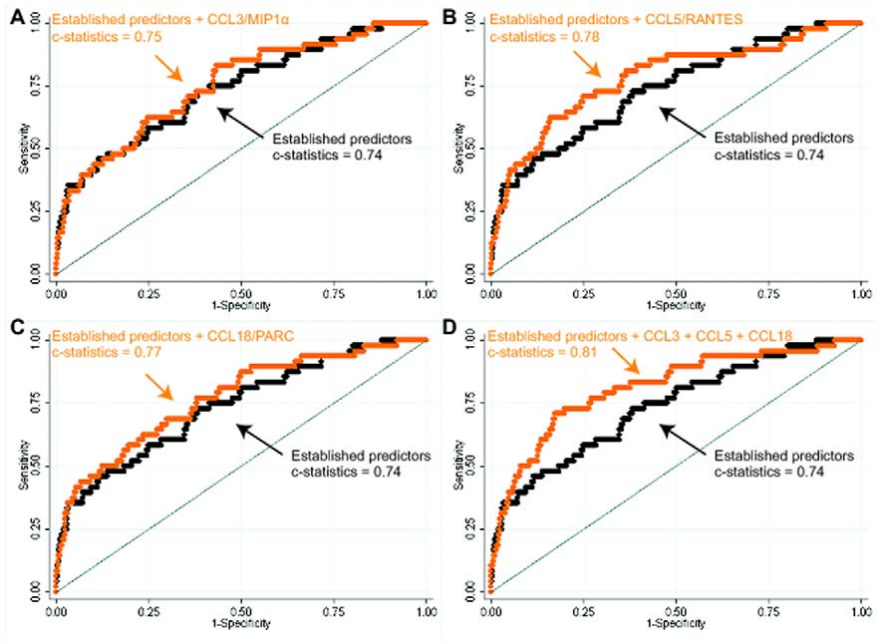
Receiver operating characteristics curves and c-statistics for logistic regression models predicting fatal events during follow-up in a subgroup of patients with acute coronary syndromes for CCL3/MIP-1α (A), CCL5/RANTES (B), CCL18/PARC (C) and all markers combined (D). Established risk predictors include age, sex, diabetes, smoking, family history of cardiovascular disease, baseline levels of NT-proBNP, CK-MB, and TnT.

Next, we examined the risk of adverse events according to the number of chemokines with concentrations in the highest tertile (table 4). Since the number of events in the patient group with all biomarkers in the lowest tertile was too small to be taken as reference group, we considered the group with at least 1 biomarker in the highest tertile as reference. For fatal and non-fatal events combined, we observed that patients with all three chemokines in the highest tertile were related to a statistically significantly increased risk of adverse events, when compared to patients with at least one chemokine in the highest tertile (HR: 2.71, 95%CI: 1.14–4.91). Patients with all chemokine levels in the lowest tertile had a decreased risk (HR: 0.49, 95%CI: 0.25–0.96). This pattern was similar for the fatal events; compared to the reference group, patients with all three chemokines in the lowest tertile were associated with a decreased risk of fatal events (HR: 0.29, 95%CI: 0.10–0.85), whereas patients with all three chemokine levels in the highest tertile were related to an increased risk (HR: 2.52, 95%CI: 1.11–5.65). Moreover, for both endpoints we observed a statistically significant trend for an increasing risk of an event with an increasing number of chemokine concentrations in the highest tertile (p≤0.018). Unfortunately, small subgroups prohibited meaningful analyses for the non-fatal events.

Based on the observed positive relationships between the chemokines and the risk of fatal events - as opposed to non-fatal events - during patient follow-up, we next investigated the markerś added prognostic value to predict the occurrence of fatal events on top of that provided by known risk predictors (figure 2). After constructing the ROC curves, the c-statistics appeared to increase only marginally when - separately - CCL3/MIP-1α, CCL5/RANTES or CCL18/PARC were added to the regression model that included known predictors of fatal events (figure 2A–C). Inclusion in the model of all three chemokines simultaneously however, increased the c-statistic statistically significantly from 0.74 to 0.81 (p = 0.007) (figure 2D).

## Discussion

High levels of CCL3/MIP1α, CCL5/RANTES and CCL18/PARC were found to be independently associated with short-term fatal events in patients with ACS. Furthermore, risk increased with an increasing number of chemokines in the highest concentration tertile. Considering known predictors of fatal events, CCL3/MIP1α, CCL5/RANTES and CCL18/PARC provided additional prognostic information only when added to the model simultaneously. In our ACS patients, no relationships were observed between chemokine levels and the risk of non-fatal events during follow-up.

Our finding that CCL3/MIP1α, CCL5/RANTES and CCL18/PARC are independent risk predictors of future fatal events confirms and extends results from two earlier studies in which we showed that CCL5/RANTES and CCL18/PARC were associated with refractory UAP [32] and CCL3/MIP1α with future ischemia events [36]. In addition, the present results indicated that the risk of follow-up events increased with an increasing number of chemokine levels in the highest tertile and that risk estimation improved when considering all three biomarkers simultaneously. The value of a multi-marker strategy has already been advocated by Zethelius and co-workers [40]. They reported an evident increase in the risk of cardiovascular mortality with an increasing number of elevated biomarker concentrations. Furthermore, several studies have reported interactions between prognostic biomarkers [41], [42], [43]. For example, levels of interleukin-10 were only predictive for death and non-fatal AMI in patients with elevated CRP levels [44]. Both these results and the current findings plead for a multi-marker strategy over a single-marker approach.

In agreement with the results of Zethelius and co-authors [40], the current findings further imply that a combination of multiple biomarkers together with established risk predictors can improve risk prediction in diseased individuals. Namely, all three biomarkers together with known predictors of fatal events in ACS patients increased the c-statistic significantly, when compared with the known risk predictors alone. Still, the question of whether this increased prognostic value is also of clinical relevance requires further investigation in studies with larger patient samples and higher numbers of events during follow-up.

Our observation that CCL3/MIP1α, CCL5/RANTES and CCL18/PARC levels were associated with the risk of fatal events, but not non-fatal events, is in line with the results of a recent study in elderly subjects with either pre-existing vascular disease or an increased risk of vascular disease due to smoking, hypertension or diabetes [45]. The authors observed that the inflammatory markers interleukin-6 and CRP were more strongly associated with the risk of fatal vascular events than with the risk of non-fatal events. In search of a possible explanation, we noticed a large difference between the timing of fatal and non-fatal events during follow-up. As the Kaplan-Meier curves showed (figure 1), the majority of the fatal events occurred already shortly after the start of follow-up. The median time-to-event of fatal events in our study was merely 5 days, whereas non-fatal events occurred much later during follow-up with a median time-to-event of 120 days. The fact that a large number of fatalities occurred early during follow-up may thus imply that high concentrations of CCL3/MIP1α, CCL5/RANTES and CCL18/PARC indicate a high risk of short-term occurring events. We observed that patients with fatal events had higher levels of CCL3/MIP1α, CCL5/RANTES and CCL18/PARC than patients with non-fatal events and appeared to have suffered a larger infarct reflected by a) a larger proportion of patients with STEMI at the time of admission (68.1% in patients with fatal events versus 36% in patients with a non-fatal event, p = 0.01), b) a higher incidence of Killip class ≥2 (0% in patients with non-fatal events and 10.6% in patients with fatal events, p = 0.09) and c) a smaller average ejection fraction of the heart (39.5% in patients with fatal events versus 48.4% in patients with non-fatal events, p = 0.01).

In the current study sample, diabetes was relatively often present in subjects that experienced an event during follow-up. Diabetes is known to be associated with increased risk of cardiovascular disease [46] and all-cause mortality [47], although the underlying mechanisms remain to be clarified. Consequently, the presence of diabetes may confound the relationship between the biomarkers under study and the risk of a future fatal or non-fatal event. Since the exclusion of all cases with diabetes would drastically decrease statistical power, we have adjusted all Cox regression models for the presence of diabetes.

A few limitations of this study should be noted. First, co-morbidities such as cancer, acute or chronic infections, and autoimmune/inflammatory diseases, were not registered for the patients included in the cohort. Possibly, certain co-morbidities may influence circulating chemokine levels. The fact that hsCRP levels were not extremely high suggests that the possible presence of infectious or inflammatory co-morbidities was low. Second, blood samples were stored for approximately 3.5 years (average storage time: 3.0±0.5 years) before measuring chemokine concentrations. It is known that long term storage and repeated freeze/thaw cycles of samples may cause blood marker degradation [48], [49], which then leads to biased results of the association between blood markers and endpoint. As such, we carefully evaluated the chemokine concentrations, storage time and time to event/censoring. Biomarker levels appeared not to have changed over time and were not correlated with event time (see figure S1). Furthermore, blood samples have not undergone repeated freeze/thaw cycles, but were used immediately upon defrosting. The average storage time was similar both patients with and without and an event during follow-up. Third, the relatively small number of events in our cohort, in particular the number of non-fatal events, warrants caution when interpreting the results of subgroup analyses. Rather than to focus on the actual size of the risk estimates, we prefer to look at the direction in which the associations are pointing. Despite low power, our results confirm those of previously published research. Future studies will need larger cohorts to validate our findings and to perform more detailed subgroup analyses to unravel the potential clinical importance of chemokines in (short-term) risk prediction in ACS patients. In addition, detailed subgroup analyses may provide clues to the underlying molecular mechanism.

In conclusion, chemokines CCL3/MIP1α, CCL5/RANTES and CCL18/PARC are independently associated with the risk of short-term fatal events in ACS patients, in which the risk increases with an increasing number of elevated chemokine concentrations. The current results plead in favour of a multi-marker strategy as opposed to a single-marker approach. The biomarkers’ added value for risk stratification lies in their combination, which improves risk prediction beyond that of a model based on known event-predictors in patients with ACS. The clinical relevance of our results for the current cardiovascular practice deserves further investigation in studies with larger patient populations.

## Supporting Information

Figure S1 **Time-to-event for acute coronary syndromes patients according to the occurrence of an event during follow-up and correlations between storage time, time-to-event and chemokine levels.** Time-to-event (days) for event free patients represents time of censoring. As to be expected, time-to-event differed between patients with and without an event during follow-up. Storage time and time-to-event correlated poorly in event-free patients (section B), and a correlation was absent in patients with an event during follow-up. Importantly, CCL3/MIP1α, CCL5/RANTES and CCL18/PARC levels had not decreased with increasing storage time in both patients with and without an event (sections C, E; and G). Likewise, we did not observe any correlation between chemokine concentrations and time-to-event (sections D, F and H).
(DOCX)
