# Supplementary figures and images for: Chemokines CCL3/MIP1α, CCL5/RANTES and CCL18/PARC are Independent Risk Predictors of Short-Term Mortality in Patients with Acute Coronary Syndromes
Source: PLoS One. 2012 Sep 21;7(9):e45804. doi: 10.1371/journal.pone.0045804 (PMC3448678; doi:10.1371/journal.pone.0045804)

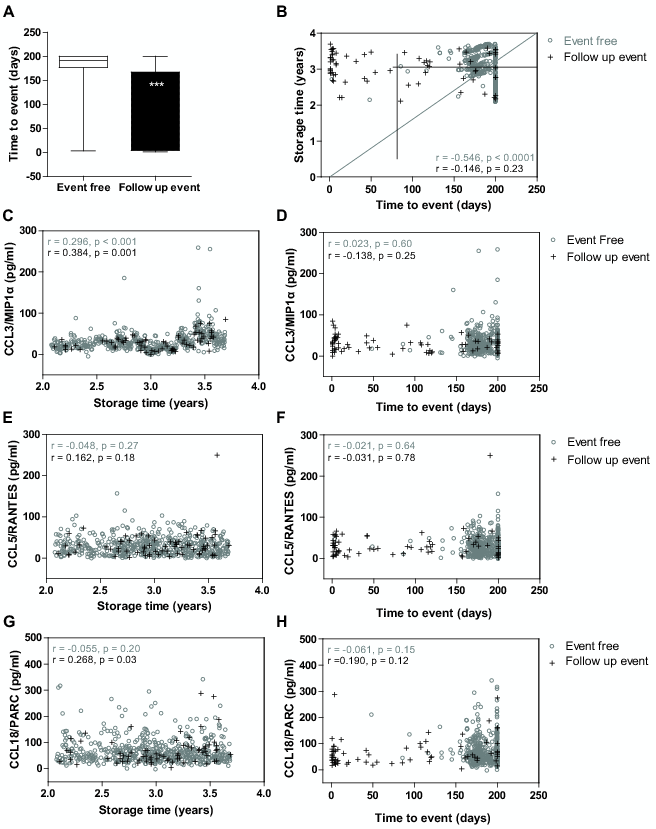

Supplement: Figure S1 — Time-to-event for acute coronary syndromes patients according to the occurrence of an event during follow-up and correlations between storage time, time-to-event and chemokine levels. Time-to-event (days) for event free patients represents time of censoring. As to be expected, time-to-event differed between patients with and without an event during follow-up. Storage time and time-to-event correlated poorly in event-free patients (section B), and a correlation was absent in patients with an event during follow-up. Importantly, CCL3/MIP1α, CCL5/RANTES and CCL18/PARC levels had not decreased with increasing storage time in both patients with and without an event (sections C, E; and G). Likewise, we did not observe any correlation between chemokine concentrations and time-to-event (sections D, F and H). (DOCX) [file pone.0045804.s001.docx]
